# Acute ACL reconstruction shows superior clinical results and can be performed safely without an increased risk of developing arthrofibrosis

**DOI:** 10.1007/s00167-019-05722-w

**Published:** 2019-09-26

**Authors:** Christoffer von Essen, Karl Eriksson, Björn Barenius

**Affiliations:** Department of Orthopaedics, Stockholm South Hospital, Karolinska Institutet, Stockholm, Sweden

**Keywords:** ACL, Reconstruction, Acute, Stiffness

## Abstract

**Purpose:**

To compare acute ACL reconstruction (ACLR) within 8 days of injury with delayed reconstruction after normalized range of motion (ROM), 6–10 weeks after injury. It was hypothesized that acute ACL reconstruction with modern techniques is safe and can be beneficial in terms of patient-reported outcomes and range of motion.

**Methods:**

The effect of acute and delayed ACLR was randomized studied on 70 patients with high recreational activity level, Tegner level 6 or more, between 2006 and 2013. Patient-reported outcomes, objective IKDC, KOOS, and manual stability measurements were documented during the 24-month follow-up period.

**Results:**

The acute ACLR group did not result in increased stiffness and showed superior outcome regarding strength and how the patient felt their knee functioning at 24 months. In addition, the acute group was not inferior to the delayed group in any assessment. Regarding patient-related outcomes in KOOS, both groups showed significant improvements in all subscales, but no difference was found between the groups. Functional return (FR) rate was almost double compared to the Swedish knee ligament register and treatment failure (TF) rate was reduced by half, no significant difference between the groups. No difference regarding cyclops removal, re-injury of ACL or meniscus was found between the two surgical timing groups.

**Conclusion:**

Acute ACLR within 8 days of injury does not appear to adversely affect ROM or result in increased stiffness in the knee joint and was not inferior to the delayed group in any assessment when compared to delayed surgery.

**Level of evidence:**

I.

## Introduction

Acute anterior cruciate ligament (ACL) rupture is a common and serious injury to the knee. The incidence in Sweden is close to 80/100,000 inhabitants, with non-contact ACL injuries most commonly occurring in athletes who participate in pivoting sports such as soccer, handball, and alpine skiing [33]. Though it has been established that ACL reconstruction (ACLR) decreases pathological knee laxity and reduces episodes of instability [13, 20, 29], many patients never return to their pre-injury activity level. In addition, irrespective of how the injury is treated, many patients later develop osteoarthritis (OA) of the knee and studies comparing long-term outcomes following ACLR compared to non-operative treatment have not demonstrated consistent results [12, 13, 16].

While it is generally agreed that ACLR is indicated for patients with signs of instability and a desire to resume a high activity level in pivoting sports, the optimal timing of ACLR has not yet been determined. The current recommendation is to delay ACLR after an acute injury due to the risk of arthrofibrosis and suboptimal clinical results [28, 37]. However, several studies have shown similar postoperative range of motion (ROM) regardless of whether surgery was performed within 48 h, 2 weeks, or if ACLR was delayed for a minimum of 6 weeks [1, 10, 17, 22, 30]. Early ACLR can facilitate early return to sport and work, and has been reported to be more cost effective, while increased time between injury and surgical intervention is associated with increased incidence of meniscus and cartilage injuries [9, 20, 23, 34].

In an initial study, involving patients with high activity levels, compared outcomes following ACLR performed within 8 days of injury to surgery 6–10-week postinjury and demonstrated no significant differences in ROM at 6-month follow-up [10]. In this study, outcomes for the two groups were assessed at a minimum of 24-month post-surgery. It was hypothesized that an acute ACLR would not result in inferior patient-reported outcomes nor a higher frequency of ROM deficits.

## Materials and methods

Active adults between 18 and 40 years of age who presented to the Orthopaedics Department of Stockholm South Hospital with an acute ACL rupture in a previously healthy knee were enrolled in the study. Exclusion criteria were: Tegner activity level [35] below level 6, major cartilage or meniscus injury on MRI requiring acute surgery, signs of OA on acute radiograph, a medial collateral ligament injury grade 2 or more, or multiple ligament injuries. During this period, 2,088 patients were assessed and 70 patients were included and randomized with the sealed envelope technique. One patient in the delayed group dropped out before surgery due to personal reasons. In an intial study, the details of recruitment process, full inclusion and exclusion criteria, patient demographics, and the randomization process have previously been published [10]. All patients provided written informed consent prior to study participation.

All reconstructions were performed using anatomical single-bundle hamstring grafts and all patients underwent a standardized rehabilitation protocol at one physiotherapy center, with full weight bearing allowed from day 1.

### Patient evaluation

Demographic data were obtained at baseline and included patient age, gender, injured side, time from injury to surgery, and concomitant injuries. Knee injury and osteoarthritis outcome score (KOOS) [26], Lysholm score [35] and Tegner activity level were obtained preoperatively. Patient-reported Tegner activity level refers prior to injury was also recorded. Preoperative examination was completed with a physical examination, including ROM (passive ROM measured with a goniometer and reported as a deficit in extension and flexion), instrumented laxity using the Rolimeter and thigh-circumference measured 10 cm proximal to the proximal pole of the patella. Follow-up examinations were performed at 6, 12, and 24 months postoperatively and included the same scores as preoperatively as well as functional strength test assessed with the single leg hop. Isokinetic peak torque strength at 60°, 180°, and 240°/s, and isometric torque strength at 60° and 180°, in both extension and flexion was measured with Biodex^®^. ACL graft failures, contralateral ACL ruptures and meniscal repair failures requiring revision meniscus surgery were recorded.

The study was approved by the regional ethics committee at the Karolinska Institute, Stockholm Sweden (reference no. 2006/404-31/3/2008/1541-32).

### Statistical analysis

Statistical analysis was performed with the IBM SPSS 25.0 software package for Macintosh. Nominal variables were tested by the *χ*^2^ test or the Fisher’s exact test. Ordinal variables and non-normally distributed interval and scale variables were evaluated by the Mann–Whitney *U* test, and the Student’s *t* test was used for normally distributed scale variables in independent groups. Longitudinal statistics were done with the paired-samples *t* test for normally distributed scale variables and the Wilcoxon signed-rank test for ordinal and non-normally distributed scale variables. The tests were two-sided. The results were considered significant at *p* < 0.05.

A sample size calculation was performed using the primary outcome variable ROM at 3 months. If the mean difference between the groups was 5° or more (corresponding to means of 122.5 vs. 117.5) and the common within-group standard deviation was 7.0, a sample size of 32 patients in each of the two groups would have a power of 80% to yield a statistically significant result, with 5% risk of a type-one error.

## Results

Demographic data of the study groups are displayed in Table 1. The only significant difference between group I (acute reconstruction) and group II (delayed reconstruction) was the time between injury and reconstruction. Despite several attempts to contact all patients by both mail and telephone, not all patients attended the 2-year clinical visit. Twelve (17%) patients were lost to follow-up, with no significant difference between groups I and II.

**Table 1:** Demographics

		Acute ACLR *n* = 33	Delayed ACLR *n* = 35	*p* value
Time injury-recon	d ± SD	5 ± 2	55 ± 8	< 0.01
OP time	min ± SD	93 ± 20	83 ± 18	n.s.
ST/Gr	*n* (%)	7 (21)	7 (20)	n.s.
Graft diameter	Mm ± SD	8.8 ± 0.8	8.6 ± 0.8	n.s.
Additional injury	*n* (%)	21 (66)	15 (47)	n.s.
Medial meniscus	*n* (%)	7 (22)	2 (6)	n.s.
Lateral meniscus	*n* (%)	13 (41)	10 (31)	n.s.
Sutures	*n* (%)	3 (9)	1 (3)	n.s.
Cartilage inj.	*n* (%)	10 (31)	4 (13)	n.s.

*ACL* anterior cruciate ligament reconstruction

Patient demographics at baseline for patients who underwent ACLR are displayed as mean ± SD, number and percentage, respectively. Statistical significant (*p* < 0.05) values were only seen for the time from injury to reconstruction

### Patient-related outcome

**Fig. 1:**
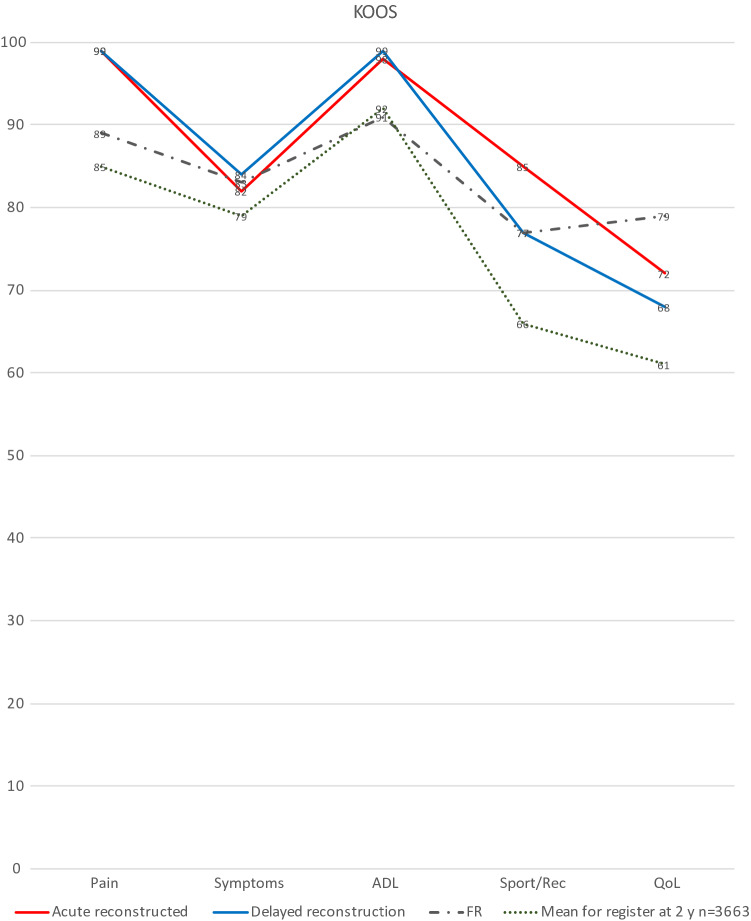
KOOS

As shown in Table 2 and Fig. 1, no difference in patient-related outcome score was found. Lysholm, KOOS, and Tegner showed no statistically significant differences between the acute and delayed treatment groups. Median Tegner level was restored to pre-injury and desired levels in both groups, with almost all patients returning to Tegner activity level 6 or higher, i.e., knee-strenuous sports.

**Table 2:** Patient-reported outcomes, instrumented knee laxity and functional strength

	Acute ACLR *n* = 28	Delayed ACLR *n* = 29	*p* value
Mean time follow-up months(SD)	25.4 (2.5)	24.7 (1.1)	n.s.
Patient-reported outcomes at 24 months			
Lysholm mean (SD)^a^			
Inclusion	32 (21.5)	43 (26.2)	n.s.
24 months	88.05 (2.4)	86.46 (2.5)	n.s.
Tegner median (range)^b^			
Before injury	8 (6–10)	9 (5–10)	n.s.
Desired	8 (6–10)	9 (5–10)	n.s.
24 months	8 (2–10)	9 (5–10)	n.s.
Return to pre-injury activity level or higher ± 1—no (%)	16 (53)	25 (86)	n.s.
Return to Tegner 6 activity level or higher—no (%)	26 (93)	28 (97)	n.s.
Instrumented knee laxity			
Rolimeter mean mm (SD)	1.8 (1.5)	2.0 (1.5)	n.s.
No (%) normal Pivot shift test^c^	28 (100)	23 (82)	n.s.
No (%) normal Lachmann test^d^	15 (54)	19 (66)	n.s.
IKDC objective score *n* (%)			
24 months			
AB	24 (88)	28 (100)	n.s.
CD	3 (12)	0 (0)	
Functional strength			
Thigh deficit circ. 10 cm above patella diff in cm (SD) ref CL	0.73 (0.94)	0.56 (1.1)	n.s.
One leg hop *n* (%)			
> 90	22 (81)	19 (66)	0.01
76–89	4 (15)	7 (24)	
50–75	0	3 (10)	
< 50	1(4)	0	
Muscle strength biodex^e^			
Ext. Isokinetic			
60°/s	94.5	89.7	n.s
180°/s	96.4	94.9	n.s
240°/s	96.2	93.8	n.s
Flex. Isokinetic			
60°/s	93.3	91.4	n.s
180°/s	96.1	88	0.05
240°/s	98.8	89.4	0.01
Ext. Isometric			
60°	96.7	95.4	n.s
180°	95.9	95	n.s
Flex. Isometric			
60°	95.4	94.5	n.s
180°	102	99.6	n.s
ROM primary endpoint^f^			
3 months			
Extension defect	3 (3.5)	2 (2.4)	n.s.
Flexion defect	7 (7.1)	6 (7.8)	n.s.
Ext. def > 5 degrees vs. CL *n* (%)	10 (31)	5 (15)	n.s.
24 months			
Extension defect	1.6 (3)	1.3 (2.5)	n.s.
Flexion defect	1.75 (2.8)	2.8 (4.1)	n.s.
Ext. def > 5 degrees vs. CL *n* (%)	4 (14)	5 (17)	n.s.
VAS question^g^			
Mean (SD)			
VAS 1			
Inclusion	83 (29)	76 (32)	n.s.
24 months	19 (21)	29 (29)	0.016
VAS 2			
Inclusion	86 (25)	82 (29)	n.s.
24 months	25 (23)	33 (30)	0.022
Functional recovery *n* (%)^h^			
24 months	12 (40)	11 (37)	n.s.
Treatment failure *n* (%)^i^			
24 months	5 (17)	7 (23)	n.s.

*ACL* anterior cruciate ligament, *CL* uninjured contralateral limb

^a^Score range from 0 to 100, with higher scores indicating better results

^b^Assesses activity level with specific emphasis on knee; scores range from 1 (least strenuous activity) to 10 (high knee demanding activity on professional sports level).15

^c^Assesses rotational stability of knee at rest result range from 0 (normal stability) to 3 (severely increased instability)

^d^Assesses stability of knee at rest result range from 0 (normal stability) to 1 (increased instability)

^e^Comparison of extensor and flexor torque deficits collected for isometric Biodex, displayed as mean percentage with reference uninjured CL set at 100

^f^Measured at the rehabilitation physiotherapy unit. Distribution of ROM between acute and delayed ACLR, displayed as mean degree defect with reference uninjured limb and SD, number and percentage, respectively

^g^VAS 1 “How does your knee function (0 (normal)-100)”, VAS 2 “How does your knee affect your activity level (0 (not at all)-100)”

^h^Defined as Knee Osteoarthritis Outcome Score (KOOS) above: 90 for Pain, 84 for Symptoms, 91 for ADL, 80 for Sport/Rec and 81 for quality of life (QoL)

^i^Defined as KOOS, QoL < 44

### Functional recovery (FR) and treatment failure (TF)

Functional recovery was defined as a Knee Osteoarthritis Outcome Score (KOOS) above: 90 for Pain, 84 for Symptoms, 91 for ADL, 80 for Sport/Rec, and 81 for quality of life (QoL). TF was defined as a KOOS, QoL < 44 [4]. No significant difference between the groups, almost 40% in each group achieved FR, was found.

### Objective IKDC and manual laxity measurements

The overall objective IKDC as well as manual laxity measurements did not display any significant differences between the acute and delayed surgery patients. It also showed high scores in both groups, with almost all patients within grade AB (normal to nearly normal), as presented in Table 2.

### Passive range of motion

The distribution of range of motion scores according to deficits in flexion and extension at 24 months in the study groups is presented in Table 2. No statistically significant difference was found between the acute and delayed treatment groups in patients with an isolated ACL tear. The patients with an extension deficit at 3-month follow-up had regained their ROM by 24 months, i.e., those found to have an extension deficit at 24 months had lost ROM subsequent to the 3-month follow-up.

### Functional strength

Similar results were found in the groups regarding muscle circumference and functional strength measured with the one leg hop test. However, significant differences were found in isokinetic flexion between groups during the Biodex test, Table 2. Values in the acute group were significantly higher for the flexor muscles at 180° and 240°/s, by 8.1% (*p* = 0.05) and 9.4% (*p* = 0.01), respectively. The other strength assessments performed were not statistically different, although higher values were found for group I.

### Additional surgery

There was additional surgery in six cases (18%) of the acute group and 13 (37%) of the delayed (n.s.), Table 3. One patient in each group sustained a graft rupture during the study period and both reported a new significant trauma. Arthroscopic removal of a cyclops lesion was necessary in 6 patients in the delayed group and one in the acute.

**Table 3:** Additional surgery

	Acute ACLR *n* = 34	Delayed ACLR *n* = 35	*p* value
Additional surgery within 24 months *n* surgeries^a^	6	13	n.s.
Reason for reoperation *n* (%)			
Cyclops lesion	1 (2.9)	6 (17.1)	
Graft rupture	1 (2.9)	1 (2.9)	
Manipulation under anesthesia	2 (5.9)	2 (5.8)	
Meniscal lesion	0	3 (8.6)	
Synovectomy	2 (5.9)	1 (2.9)	

*ACLR* anterior cruciate ligament reconstruction

^a^Number of surgeries, not patients

## Discussion

The most important finding of this randomized control trial is that good clinical results can be achieved 24 months after acute ACLR and that early extension deficits seen at the 3-month follow-up had resolved.

This study supports the findings of other recent studies which have demonstrated that the timing of ACLR does not influence postoperative ROM [1, 7, 10, 17, 24] and contradicts the findings of older studies including Shelbourne et al. [25, 28, 37]. These differences may be due to the fact that these older studies were performed without the use of contemporary arthroscopic techniques, were retrospective, and perhaps most importantly had a more restrictive postoperative rehabilitation regime. In addition, a lack of classification in the literature regarding which timeframe constitutes acute vs. delayed surgery makes it difficult to compare these studies [11]. In this study, acute reconstruction did not result in increased stiffness. There were superior outcomes for the acute group regarding strength and how the patient perceived their knee function at 24 months. In addition, the acute group was not inferior to the delayed group in any assessment. Regarding patient-related outcomes in KOOS, both groups showed significant improvements in all subscales, but no difference was found between the groups. The KOOS results were also slightly better than those from the Swedish anterior cruciate ligament registry [2] and the results from a US cohort study [32]. This further supports the fact that acute ACLR is a safe option.

Non-operative treatment of an ACL injury remains an option and can yield satisfactory results [13, 15, 31], although the body of evidence is limited due to the scarcity of randomized studies. Frobell et al. concluded that for adults with acute ACL injuries, there is no difference between surgical management (ACL reconstruction followed by structured rehabilitation) and non-operative treatment (structured rehabilitation only) in patient‐reported outcomes of knee function at 2 and 5 years after injury. However, nearly, 50% of the participants with an ACL rupture remained symptomatic following rehabilitation and later opted for ACL reconstruction surgery [13].

An association between the time from injury to surgery and the risk of additional medial meniscal injuries and chondral injuries has been reported by several studies investigating ACL injuries [4–6, 19]. Chhadia et al. found a significant association between medial meniscal injury, as well as medial and lateral compartment chondral injury, and delayed surgery (beyond 6 and 12 months) [8]. With the increased risk for further injuries, it is questionable whether initial non-operative treatment in patients with a high pre-injury activity level is the best alternative.

Anterior cruciate ligament reconstruction is commonly recommended for patients who participate in pivoting sports and aspire to return to pre-injury sports participation. The rates of return to pre-injury level of sport following ACLR differ between studies and between elite and non-elite athletes (83% vs. 63%) [3, 21]. Using pre-injury level of sport as a measure of successful surgery may not accurately reflect positive outcomes, for example, patients with pre-injury Tegner levels of ten who subsequently return to a score of eight. The study confirms that while not all patients did return to pre-injury level of sport, almost all could return to a knee-strenuous sport. No significant difference between the groups in this regard was found, but it was noted that fewer had returned in the acute group. A further in-depth analysis of these acutely operated patients who did not return to knee-strenuous sport found that they did not demonstrate inferior outcomes in any subjective or objective assessment when compared to patients who returned to their previous activity level. Furthermore, the FR and TR did not differ between those who returned and those who did not. KOOS also showed higher score in sport/req, even though fewer returned to the same Tegner levels. As such, it is unclear why they did not return to their previous level. As this study was not designed specifically to look at return to sport, one explanation of this finding could be a type 2 error, due to too few patients being included in the study to be able to accurately assess return to sport outcomes.

The ACL graft failure rate in this study was low and did not differ between the groups (2.9%) [14, 18].

The FR rate was almost double that seen in a previous study found in the Swedish Knee Ligament Register (SKLR) [4]. Although waiting time itself does not have an impact on the outcome, it can be argued that an acute or early ACLR, which is performed before recurrent giving ways occur, increases the likelihood of achieving FR. The study also showed that TF was reduced by half in the acute group compared to the SKLR. This suggests early surgery, before recurring giving ways have occurred, decreases additional injuries, and increases the likelihood of FR. Finally, it should be mentioned that an acute ACLR has been shown to be more cost-effective than delayed surgery, and time spent on rehabilitation before the surgery is better utilized postoperatively [23, 27, 36].

The major strength of this study is its prospective, randomized design and the use of the same surgical technique. Furthermore, one center with the same postoperative rehabilitation protocol was used in both groups. The two groups were also comparable in terms of age, gender and pre-injury Tegner activity level, factors which could contribute to selection bias in a non-randomized trial.

Potential limitations are the limited number of patients. Though there were sufficient numbers according to the power analysis for the primary endpoint, there may not have been enough patients included to detect other significant differences. A further limitation was the change in surgical method during the study period (transtibial vs. femoral portal drilling). Furthermore, there were a relative high number of patients who were lost to the latest follow-up, although with no difference between the groups.

## Conclusion

This study provides further evidence that acute ACL reconstruction can be performed safely without an increased risk of developing stiffness. Thus, clinicians can make their decision about the optimal time for surgery for each individual patient based on other parameters and plan acute reconstruction if indicated.
