# Correction to: Acute ACL reconstruction shows superior clinical results and can be performed safely without an increased risk of developing arthrofibrosis

**DOI:** 10.1007/s00167-021-06500-3

**Published:** 2021-04-18

**Authors:** Christoffer von Essen, Karl Eriksson, Björn Barenius

**Affiliations:** Department of Orthopaedics, Stockholm South Hospital, Karolinska Institutet, Stockholm, Sweden

## Correction to: Knee Surgery, Sports Traumatology, Arthroscopy (2020) 28:2036–2043 10.1007/s00167-019-05722-w

In the article entitled ‘Acute ACL reconstruction shows superior clinical results and can be performed safely without an increased risk of developing arthrofibrosis’ (Knee Surg Sports Traumatol Arthrosc. 2020 Jul;28(7):2036-2043.doi: 10.1007/s00167-019-05722-w. Epub 2019 Sep 26.), by von Essen et al., there was an error on page 2039, Table 2. Specifically, in the section of the table stated Return to pre-injury activity level. The table report the rate for returning to pre-injury activity level as not statistically significant between the acute and delayed groups, although it is. Unfortunately, one line of the table is missing, and therefore the n.s. is stated where is should not be.

The table should read:

**Table:** 

Return to preinjury activity level: no (%)	16 (57)	25 (86)	**0.02**
Return to preinjury activity level or higher ±1: no (%)	24 (88)	28 (97)	n.s
Return to Tegner 6 activity level or higher: no (%)	26 (93)	28 (97)	**n.s.**

